# Supplementary figures and images for: Whole Brain Mapping of Orexin Receptor mRNA Expression Visualized by Branched In Situ Hybridization Chain Reaction
Source: eNeuro. 2024 Feb 6;11(2):ENEURO.0474-23.2024. doi: 10.1523/ENEURO.0474-23.2024 (PMC10883752; doi:10.1523/ENEURO.0474-23.2024)

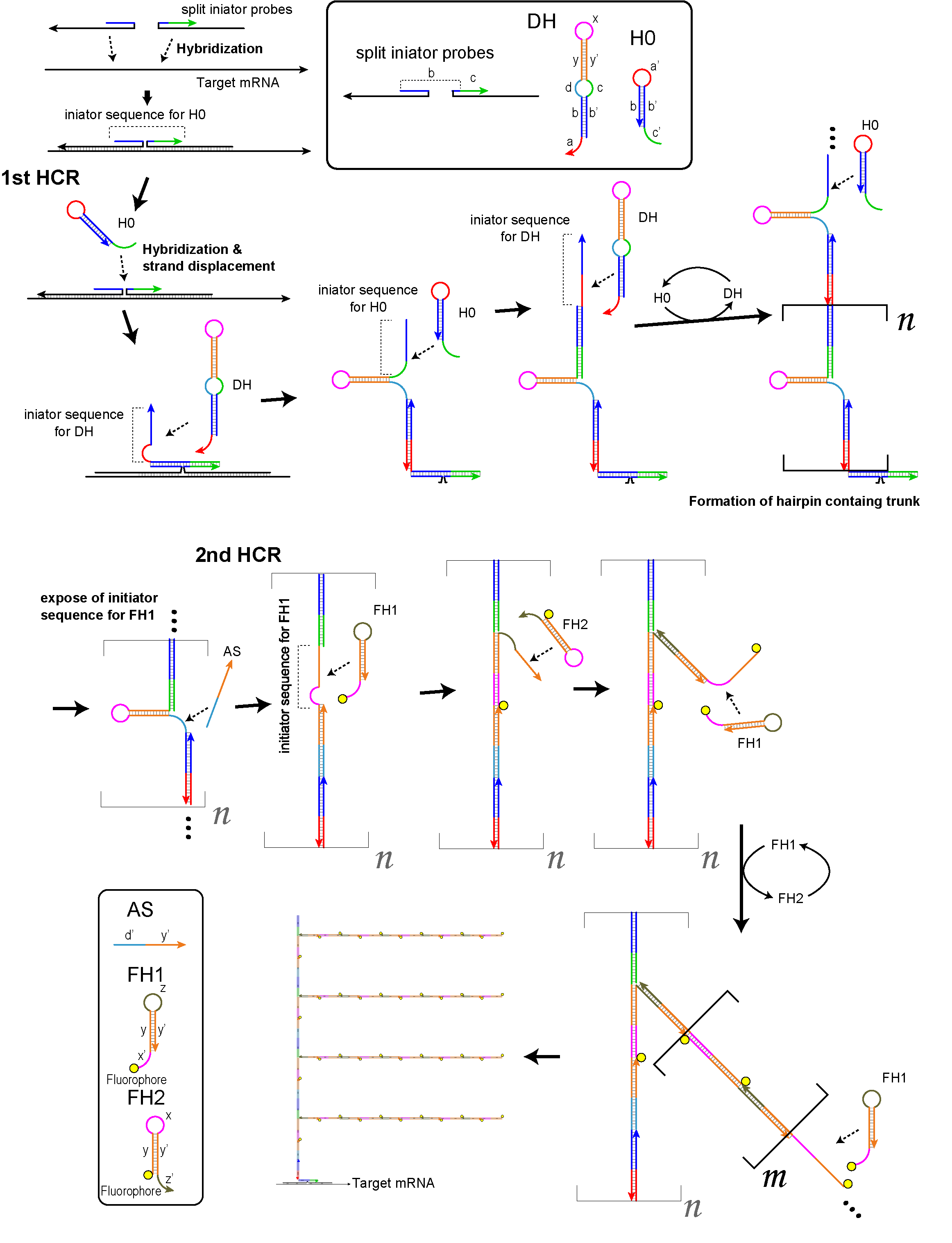

Supplement: Figure 1-1 — Principle of branched in situ hybridization chain reaction (HCR) Split-initiator probes hybridize with the target mRNA to form an initiator sequence (bc) for hairpin DNA H0. In the 1st HCR, H0 hybridizes with the pair of split-initiator probes, followed by exposure of initiator sequence (a'b') for double stem-hairpin DH. DH hybridizes with the 3' end of H0, and the initiator sequence for H0 is exposed from DH. As a consequence of HCR, the DNA trunk with the repeated hairpin-containing domain is formed on the target mRNA. In the 2nd HCR, the assist oligo (AS) hybridizes with the hairpin-containing domain to expose the initiator sequence (xy') of fluorescent hairpin DNA FH1. FH1 hybridizes with the single-strand region of the trunk to form branched DNA with the initiator sequence (zy) of FH2. HCR of FH1 and FH2 occur in multiple regions of the DNA trunk, enabling higher polymerization of fluorescent hairpins per single target site than non-branched HCR. Download Figure 1-1, TIF file. [file eneuro-11-ENEURO.0474-23.2024-s002.tif]

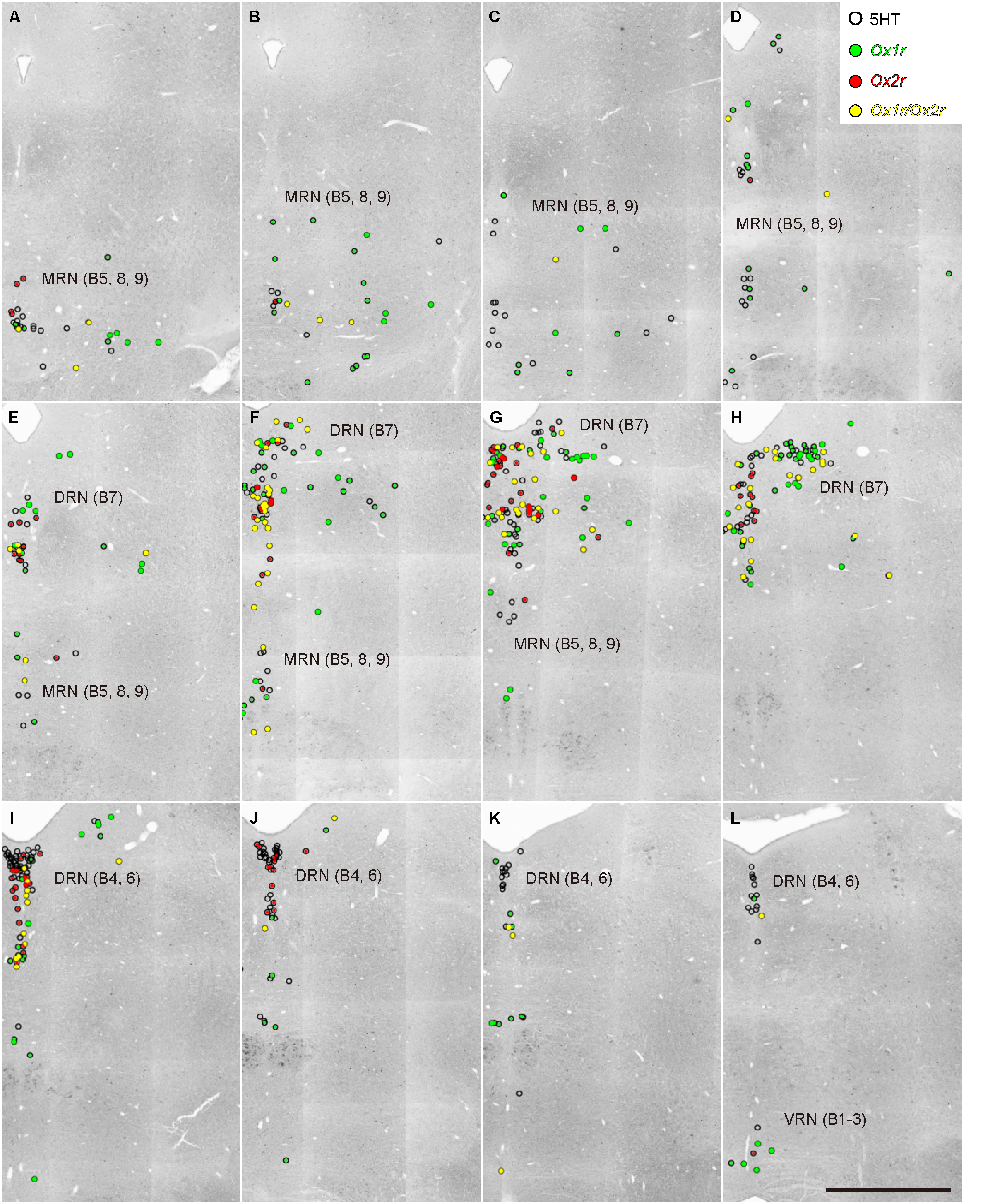

Supplement: Figure 22-1 — Distribution of orexin receptor-expressing serotonergic neurons (1/2). White, green, red, and yellow circles indicate receptor-negative, Ox1r-positive, Ox2r-positive, and both Ox1r and Ox2r-positive serotonergic neurons, respectively. Panels are arranged in anterior-posterior order. Scale bar: 500 μm. Download Figure 22-1, TIF file. [file eneuro-11-ENEURO.0474-23.2024-s003.tif]

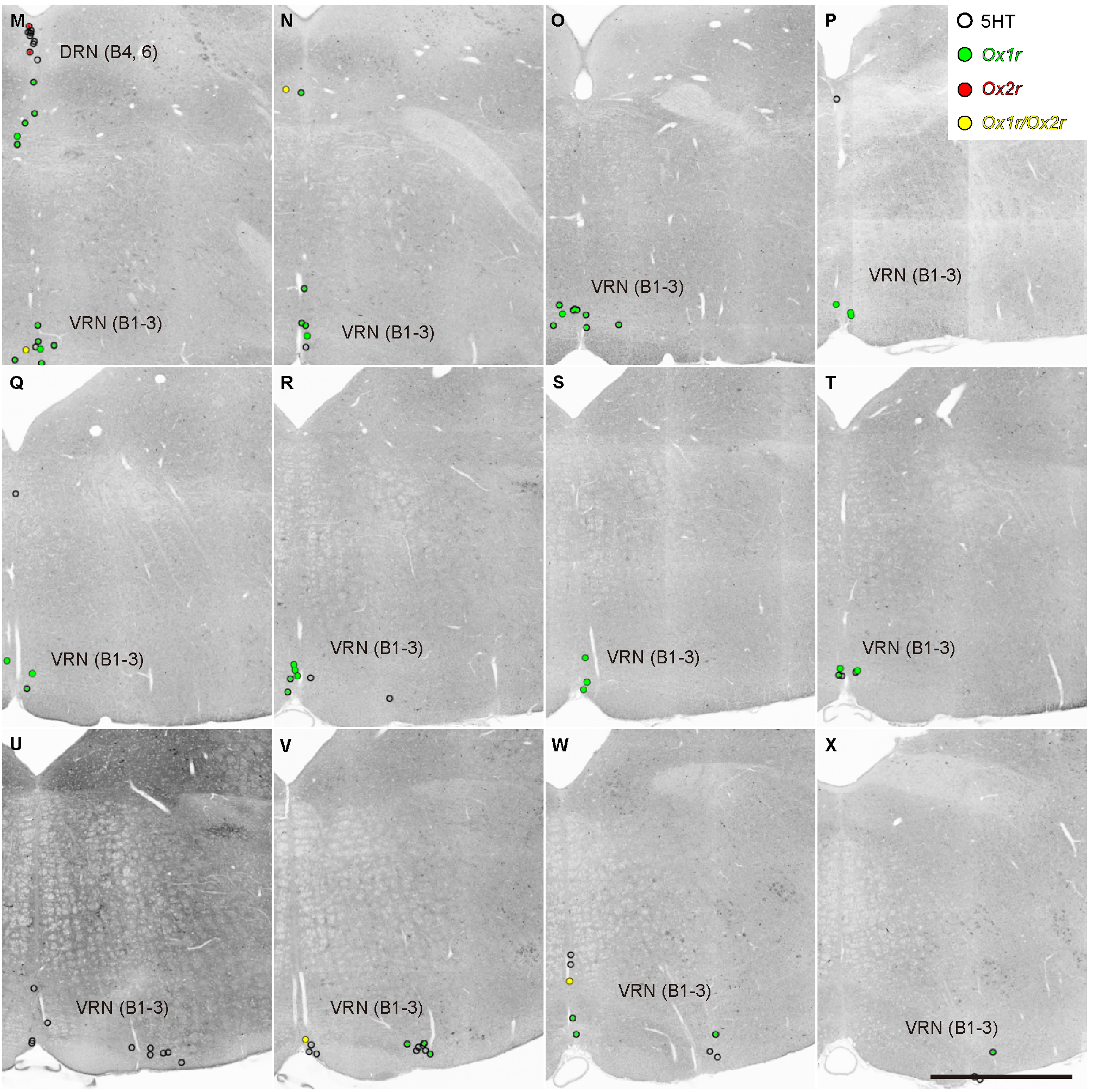

Supplement: Figure 22-2 — Distribution of orexin receptor-expressing serotonergic neurons (2/2). White, green, red, and yellow circles indicate receptor-negative, Ox1r-positive, Ox2r-positive, and both Ox1r and Ox2r-positive serotonergic neurons, respectively. Panels are arranged in anterior-posterior order. Scale bar: 500 μm. Download Figure 22-2, TIF file. [file eneuro-11-ENEURO.0474-23.2024-s004.tif]

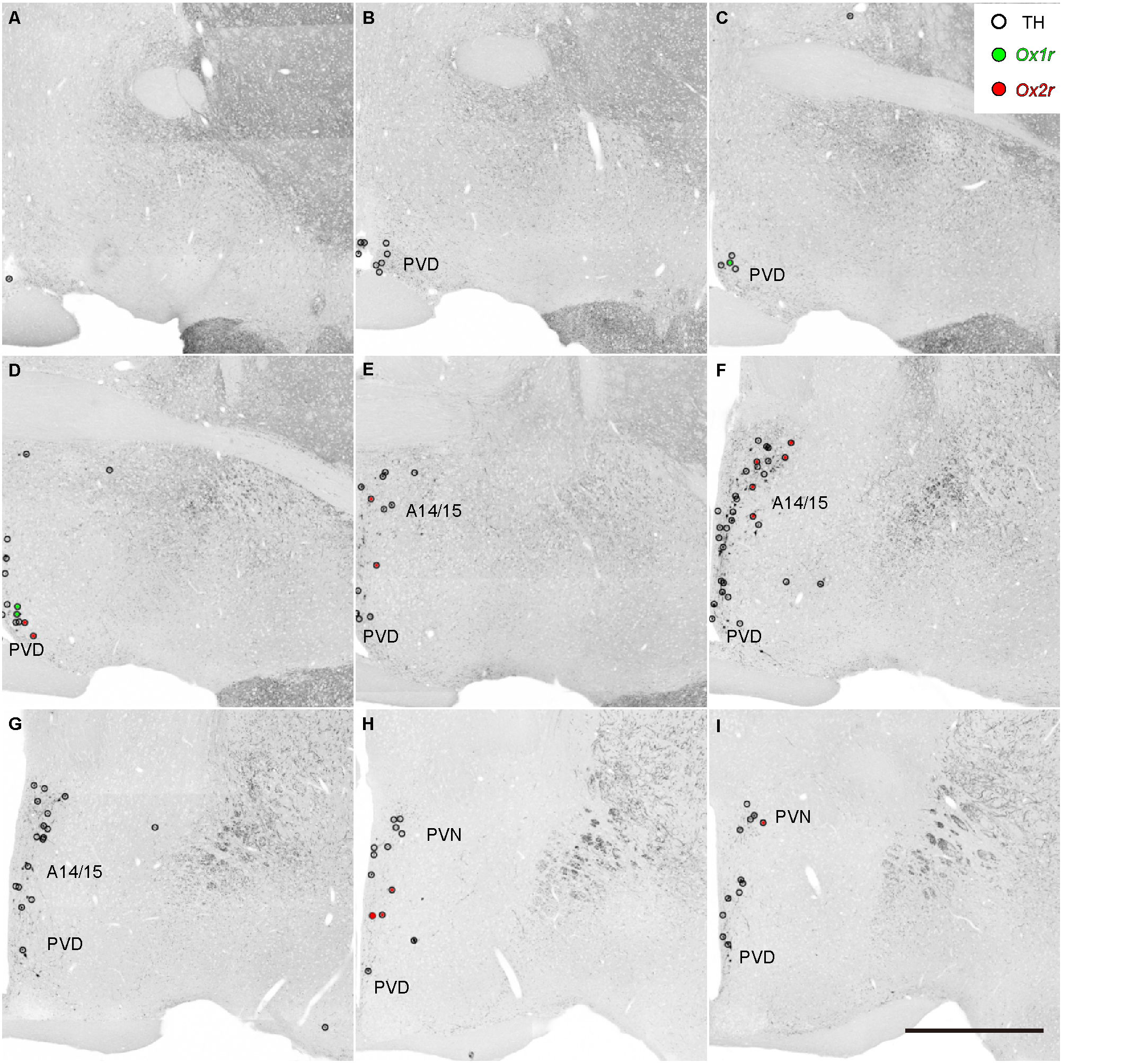

Supplement: Figure 23-1 — Distribution of orexin receptor-expressing dopaminergic/adrenergic neurons (1/6). White, green and red circles indicate receptor-negative, Ox1r-positive, and Ox2r-positive dopaminergic/adrenergic neurons, respectively. Panels are arranged in anterior-posterior order. Scale bar: 500 μm. Download Figure 23-1, TIF file. [file eneuro-11-ENEURO.0474-23.2024-s005.tif]

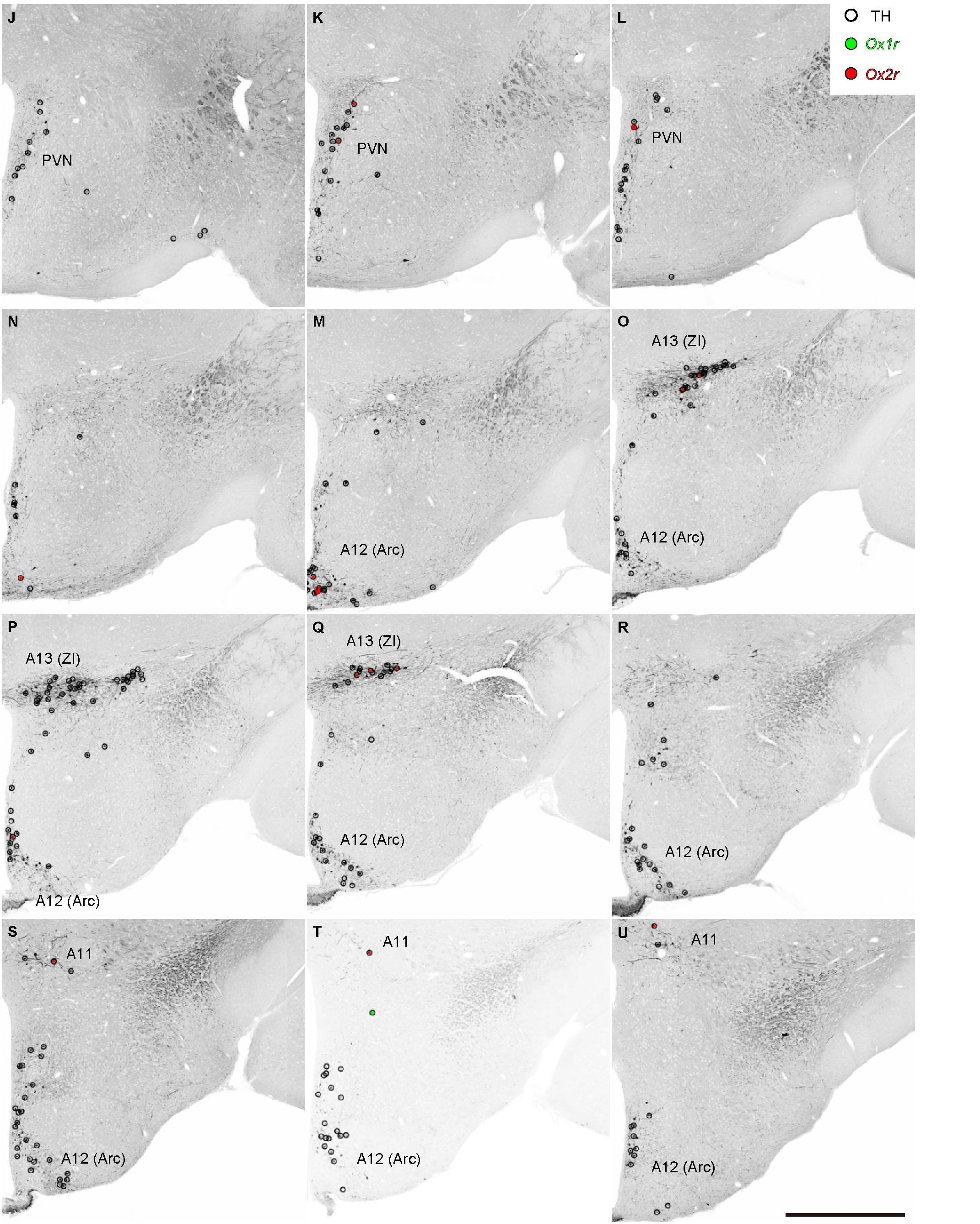

Supplement: Figure 23-2 — Distribution of orexin receptor-expressing dopaminergic/adrenergic neurons (2/6). White, green and red circles indicate receptor-negative, Ox1r-positive, and Ox2r-positive dopaminergic/adrenergic neurons, respectively. Panels are arranged in anterior-posterior order. Scale bar: 500 μm. Download Figure 23-2, TIF file. [file eneuro-11-ENEURO.0474-23.2024-s006.tif]

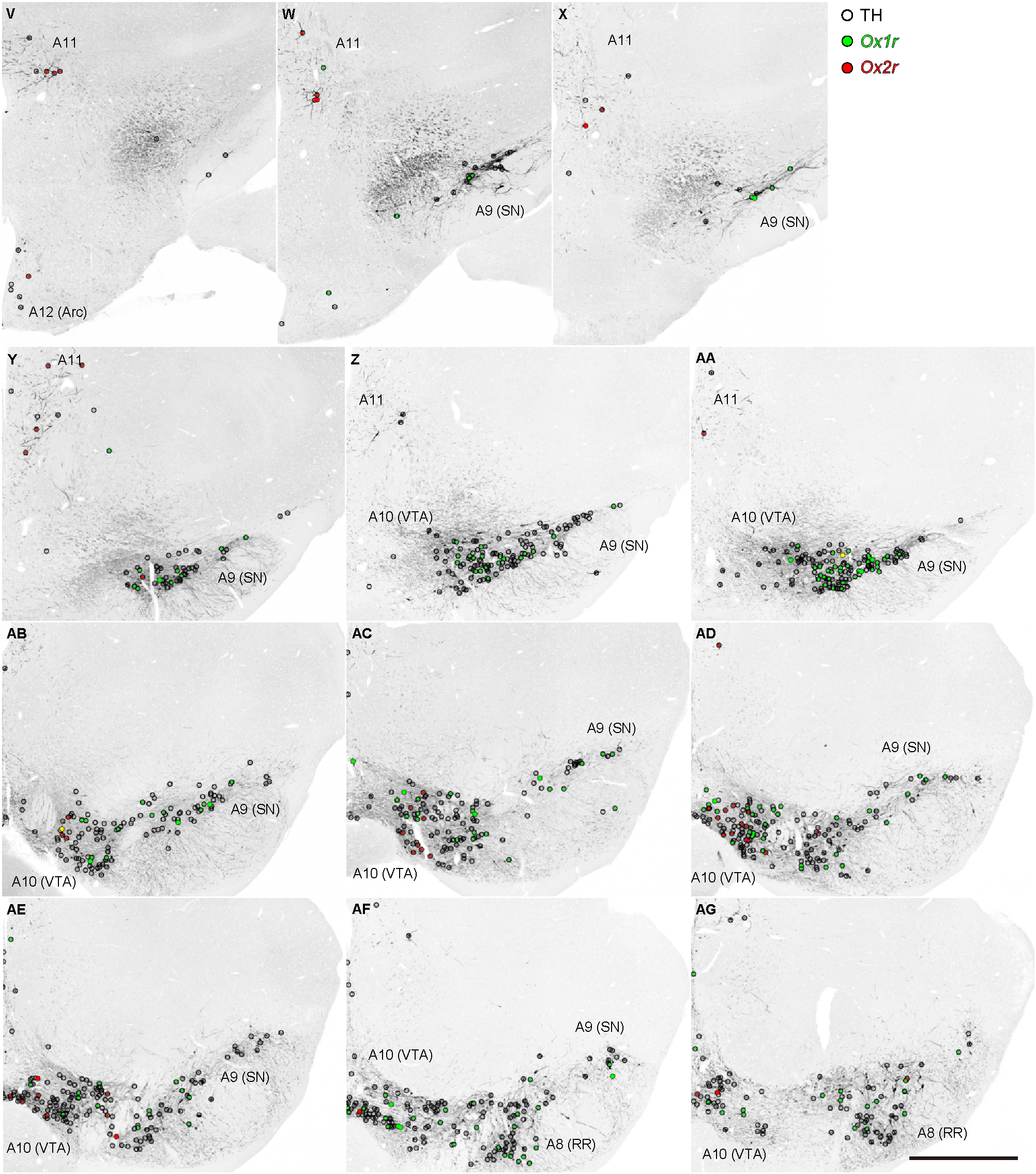

Supplement: Figure 23-3 — Distribution of orexin receptor-expressing dopaminergic/adrenergic neurons (3/6). White, green and red circles indicate receptor-negative, Ox1r-positive, and Ox2r-positive dopaminergic/adrenergic neurons, respectively. Panels are arranged in anterior-posterior order. Scale bar: 500 μm. Download Figure 23-3, TIF file. [file eneuro-11-ENEURO.0474-23.2024-s007.tif]

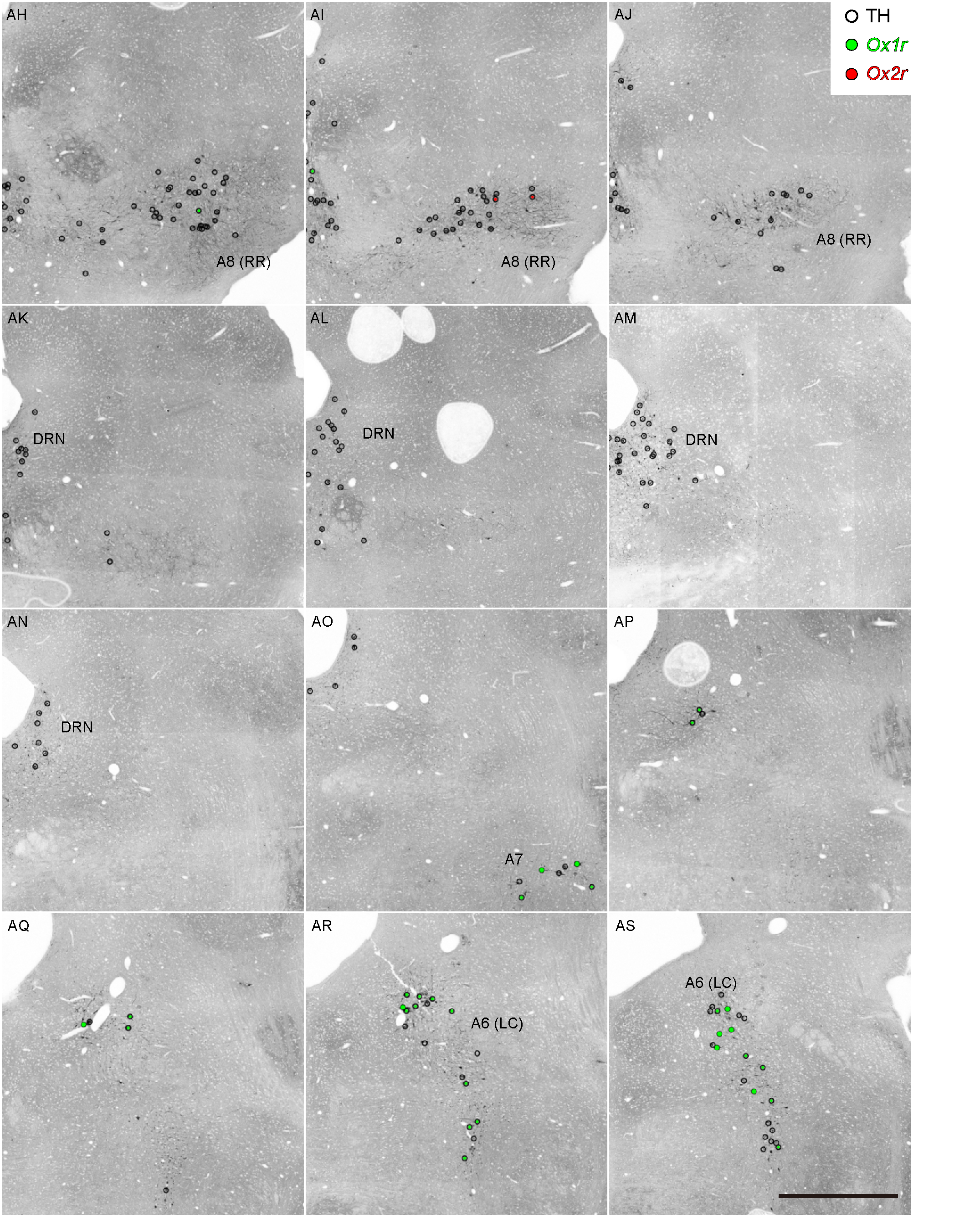

Supplement: Figure 23-4 — Distribution of orexin receptor-expressing dopaminergic/adrenergic neurons (4/6). White, green and red circles indicate receptor-negative, Ox1r-positive, and Ox2r-positive dopaminergic/adrenergic neurons, respectively. Panels are arranged in anterior-posterior order. Scale bar: 500 μm. Download Figure 23-4, TIF file. [file eneuro-11-ENEURO.0474-23.2024-s008.tif]

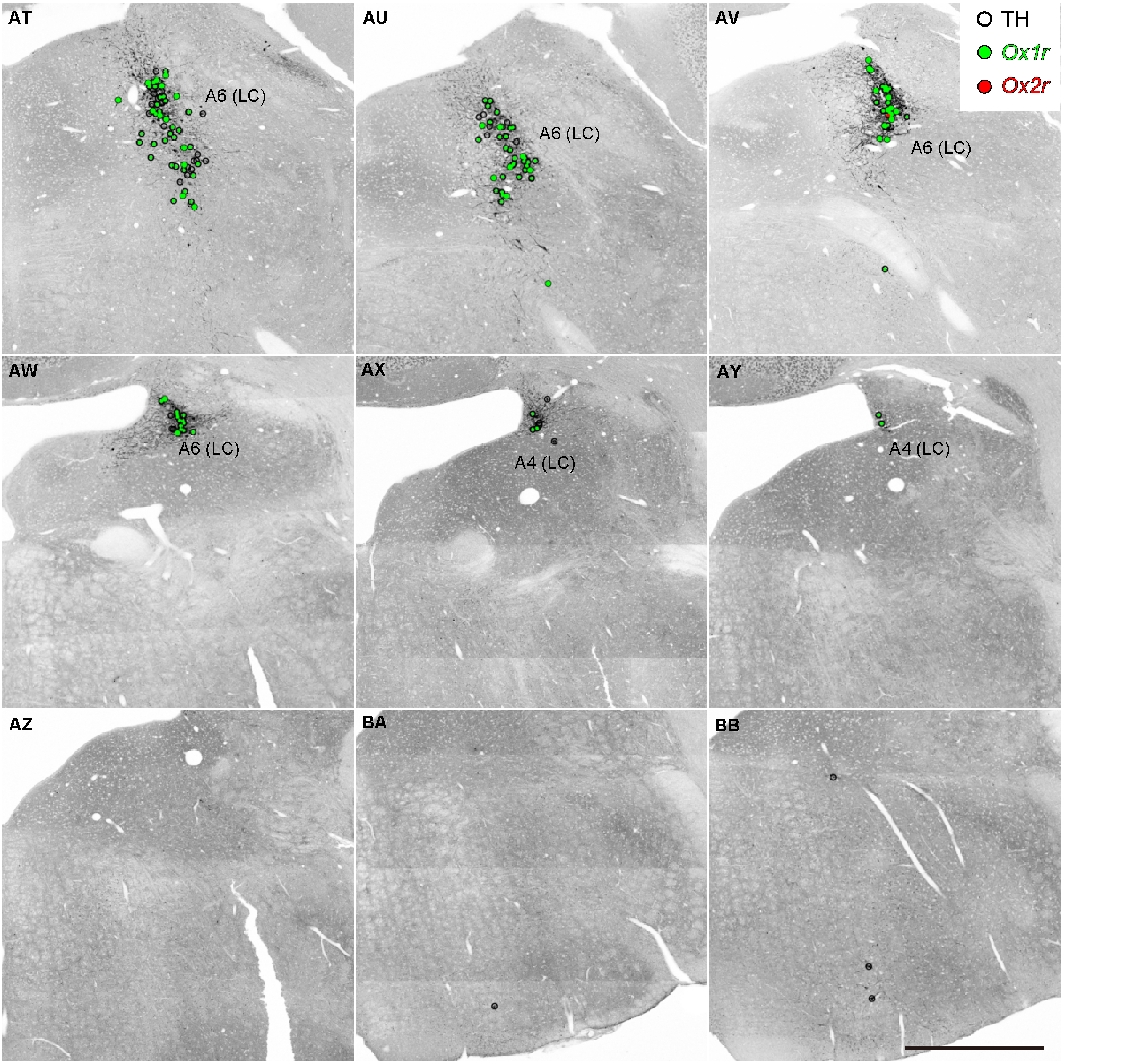

Supplement: Figure 23-5 — Distribution of orexin receptor-expressing dopaminergic/adrenergic neurons (5/6). White, green and red circles indicate receptor-negative, Ox1r-positive, and Ox2r-positive dopaminergic/adrenergic neurons, respectively. Panels are arranged in anterior-posterior order. Scale bar: 500 μm. Download Figure 23-5, TIF file. [file eneuro-11-ENEURO.0474-23.2024-s009.tif]

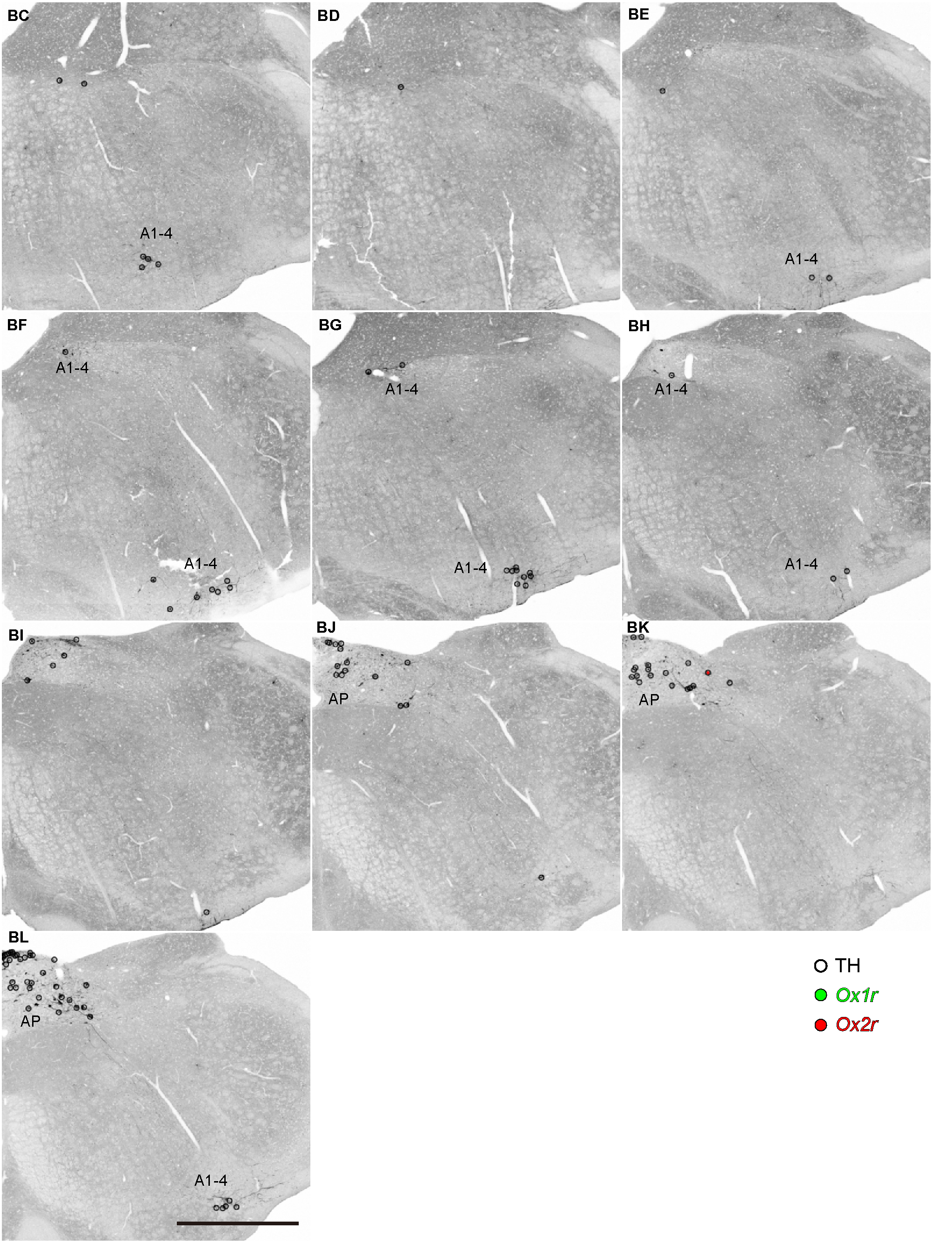

Supplement: Figure 23-6 — Distribution of orexin receptor-expressing dopaminergic/adrenergic neurons (6/6). White, green and red circles indicate receptor-negative, Ox1r-positive, and Ox2r-positive dopaminergic/adrenergic neurons, respectively. Panels are arranged in anterior-posterior order. Scale bar: 500 μm. Download Figure 23-6, TIF file. [file eneuro-11-ENEURO.0474-23.2024-s010.tif]

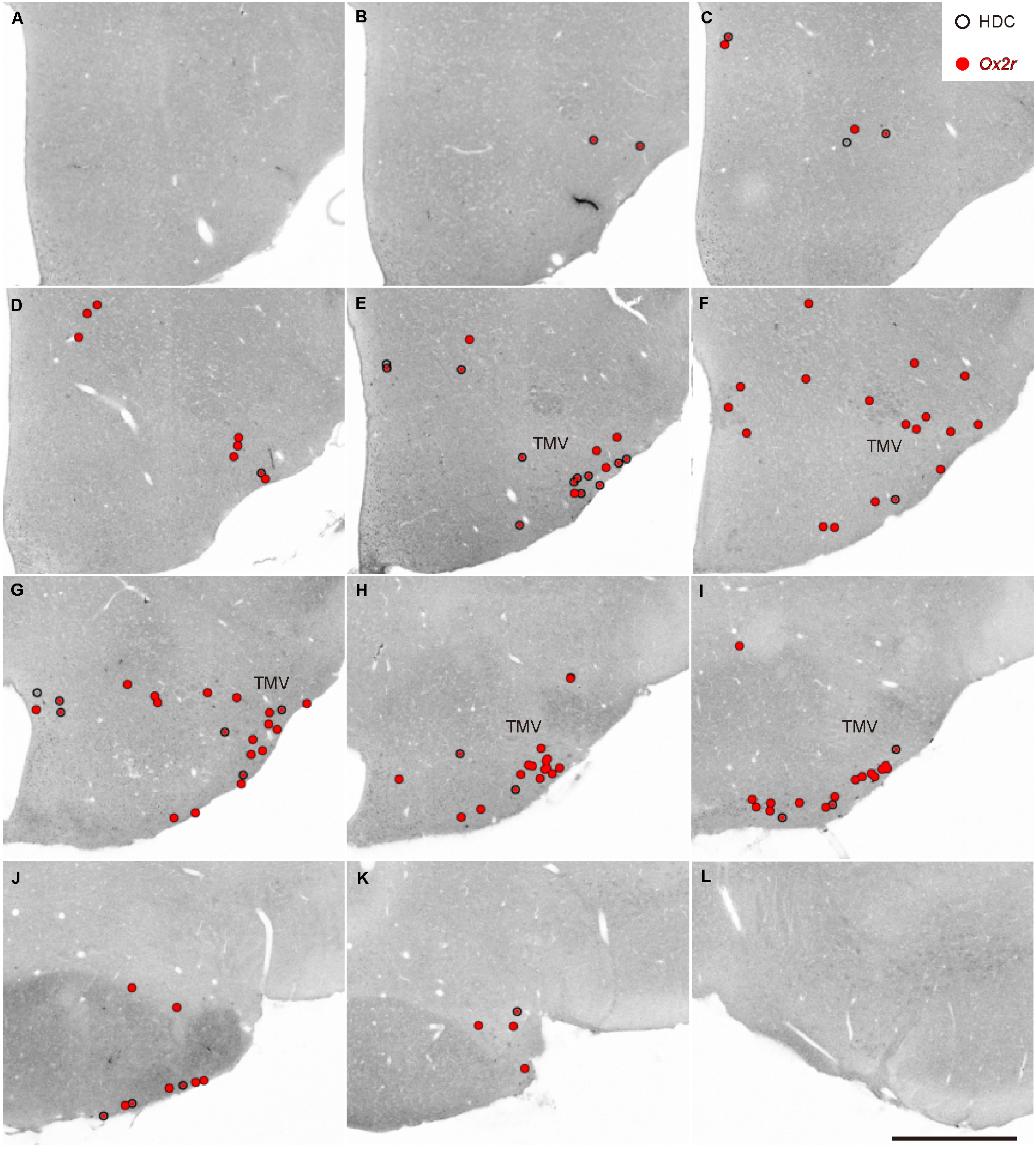

Supplement: Figure 23-7 — Distribution of Ox2r-expressing histaminergic neurons White and red circles indicate Ox2r-negative and Ox2r-positive histaminergic neurons, respectively. Panels are arranged in anterior-posterior order. Scale bar: 500 μm. Download Figure 23-7, TIF file. [file eneuro-11-ENEURO.0474-23.2024-s011.tif]

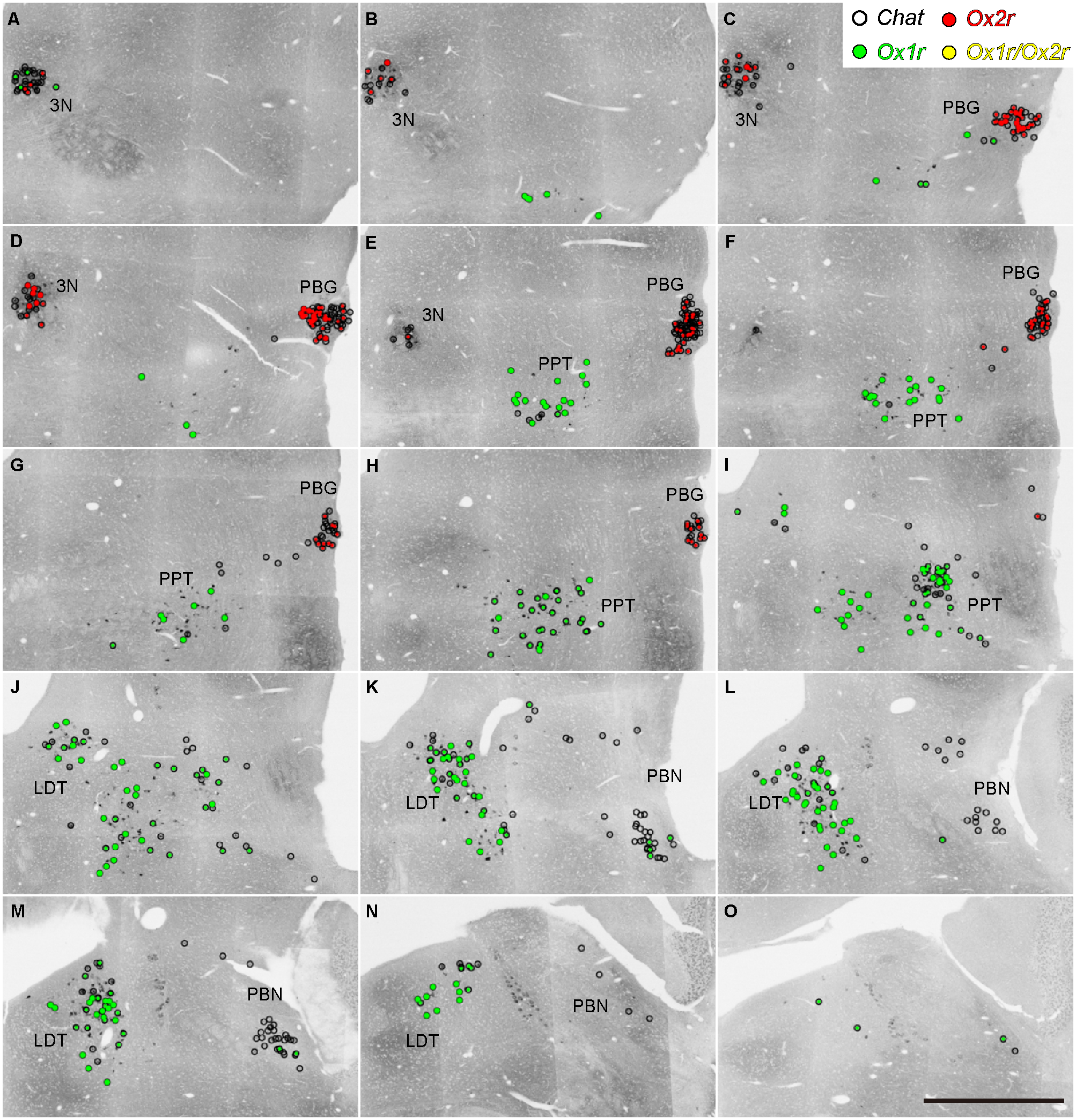

Supplement: Figure 24-1 — Distribution of orexin receptor-expressing cholinergic neurons in the brainstem (1/2). White, green, red, and yellow circles indicate receptor-negative, Ox1r-positive, Ox2r-positive, and both Ox1r- and Ox2r-positive cholinergic neurons, respectively. Panels are arranged in anterior-posterior order. Scale bar: 500 μm. Download Figure 24-1, TIF file. [file eneuro-11-ENEURO.0474-23.2024-s012.tif]

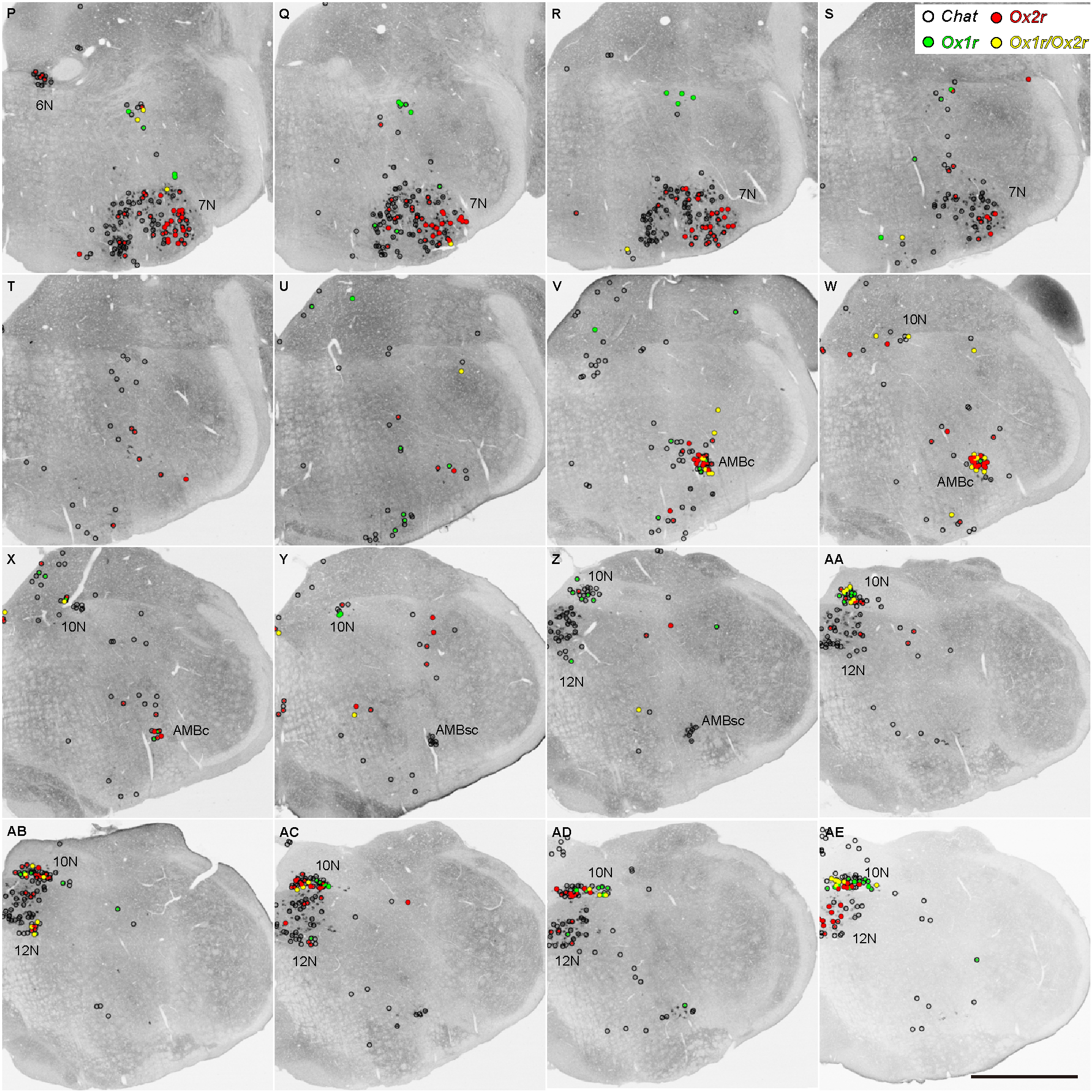

Supplement: Figure 24-2 — Distribution of orexin receptor-expressing cholinergic neurons in the brainstem (2/2). White, green, red, and yellow circles indicate receptor-negative, Ox1r-positive, Ox2r-positive, and both Ox1r- and Ox2r-positive cholinergic neurons, respectively. Panels are arranged in anterior-posterior order. Scale bar: 500 μm. Download Figure 24-2, TIF file. [file eneuro-11-ENEURO.0474-23.2024-s013.tif]
